# Chronic consumption of a hypercaloric diet increases neuroinflammation and brain senescence, promoting cognitive decline in middle-aged female Wistar rats

**DOI:** 10.3389/fnagi.2023.1162747

**Published:** 2023-04-17

**Authors:** Verónica Salas-Venegas, Roberto Santín-Márquez, Ricardo Jair Ramírez-Carreto, Yesica María Rodríguez-Cortés, Agustina Cano-Martínez, Armando Luna-López, Anahí Chavarría, Mina Konigsberg, Norma Edith López-Díazguerrero

**Affiliations:** ^1^Posgrado en Biología Experimental, Universidad Autónoma Metropolitana-Unidad Iztapalapa, Mexico City, Mexico; ^2^Departamento de Ciencias de la Salud, División de Ciencias Biológicas y de la Salud (DCBS), Universidad Autónoma Metropolitana Iztapalapa, CDMX, Mexico City, Mexico; ^3^Programa de Doctorado en Ciencias Biomédicas, Universidad Nacional Autónoma de México, CDMX, Mexico City, Mexico; ^4^Unidad de Investigación en Medicina Experimental, Facultad de Medicina, Universidad Nacional Autónoma de México, CDMX, Mexico City, Mexico; ^5^Departamento de Fisiología, Instituto Nacional de Cardiología “Ignacio Chávez”, CDMX, Mexico City, Mexico; ^6^Departamento de Investigación Básica, Instituto Nacional de Geriatría, CDMX, Mexico City, Mexico

**Keywords:** obesity, female, neuroinflammation, senescence, cognitive deterioration

## Abstract

Being overweight and obesity are world health problems, with a higher prevalence in women, defined as abnormal or excessive fat accumulation that increases the risk of chronic diseases. Excess energy leads to adipose expansion, generating hypertrophic adipocytes that produce various pro-inflammatory molecules. These molecules cause chronic low-intensity inflammation, affecting the organism’s functioning and the central nervous system (CNS), inducing neuroinflammation. The neuroinflammatory response during obesity occurs in different structures of the CNS involved in memory and learning, such as the cortex and the hippocampus. Here we analyzed how obesity-related peripheral inflammation can affect CNS physiology, generating neuroinflammation and promoting cellular senescence establishment. Since some studies have shown an increase in senescent cells during aging, obesity, and neurodegenerative diseases, we proposed that cellular senescence participation may contribute to the cognitive decline in an obesity model of middle-aged female Wistar rats. The inflammatory state of 6 and 13 months-old female Wistar rats fed with a hypercaloric diet was measured in serum and CNS (cortex and hippocampus). Memory was evaluated using the novel object recognition (NOR) test; the presence of senescent markers was also determined. Our data suggest that the systemic inflammation generated by obesity induces a neuroinflammatory state in regions involved in learning and memory, with an increase in senescent markers, thus proposing senescence as a potential participant in the negative consequences of obesity in cognition.

## Introduction

Overweight and obesity are defined as abnormal or excessive fat accumulation that can harm health (World Health Organization, 2023), increasing the risk of developing different metabolic disorders and diseases (Awada et al., 2013), such as type 2 diabetes, cardiovascular diseases (Haslam and James, 2005), musculoskeletal disorders, and deterioration in cognitive function (Miller and Spencer, 2014). Interestingly, obesity is more common in women than men, independent of age, geographic region, or socioeconomic status (Kroll et al., 2020).

During obesity, the energy excess leads to adipose expansion generating hypertrophic adipocytes that produce a wide variety of proinflammatory molecules, such as monocyte chemoattractant protein-1 (MCP-1), TNF-α, IL-1β, and IL-6 that activate and attract the immune system cells (McArdle et al., 2013). The production of these molecules by adipocytes generates a chronic, low-intensity inflammation, affecting the organism’s functioning; the central nervous system (CNS) may also be affected. Studies performed in rodent models show that obesity causes neuroinflammation (Miller and Spencer, 2014; Li et al., 2018; Zhou et al., 2020; Salas-Venegas et al., 2022). A hypercaloric diet induces the blood–brain barrier (BBB) loss of integrity, allowing proinflammatory cytokines to enter the CNS and promoting peripheral macrophage infiltration to the brain, which subsequently contributes, among other factors such as increased peripheral free fatty acid circulation, contributing to obesity-associated neuroinflammation (Samara et al., 2020). The inflammation in specific brain regions is related to memory impairment with significant cognitive decline (Palavra et al., 2016; Castro et al., 2017; Noble et al., 2017). Intriguingly, hippocampal neuroinflammation causes deficits in memory tasks in rodent models of obesity (Samara et al., 2020).

Inflammatory responses can be beneficial as acute, transient reactions to harmful conditions, promoting the defense, repair, and adaptation of host tissues. However, chronic and low-grade inflammation is detrimental to many tissues and likely to hamper normal functions (Calder et al., 2017). Chronic inflammation has become a hallmark of neurodegeneration in the brain, with persistent microglial activation, increased proinflammatory cytokines, and elevated levels of oxidative stress (Azam et al., 2021).

Additionally, some results indicate that the production of pro-inflammatory cytokines significantly reduces the expression of brain-derived neurotrophic factor (BDNF), the most expressed neurotrophin in the brain that plays a crucial role in learning and memory. The decreased BDNF expression has been associated with cognitive dysfunction and dementia (Budni et al., 2015). BDNF favors cognitive function by increasing neurogenesis, neuronal survival, axonal growth, dendritic growth, synaptic plasticity, neuronal development, and maintenance in the central nervous system neurons (Sandrini et al., 2018).

Moreover, clinical studies have shown that obesity increases the risk of developing mild cognitive impairment in short-term memory and executive function (Nguyen et al., 2014); however, the precise mechanisms are still unknown.

In addition, it has been reported that organisms fed with high-fat diets present an accumulation of senescent cells in tissues such as the prostate, heart, and adipose tissue with implications for the inflammatory state (Wang et al., 2009; Tikoo et al., 2017). Recently, Bussian et al. (2018) reported that the accumulation of senescent astrocytes and microglia increased neurodegeneration and cognitive decline. They observed that eliminating senescent cells prevented reactive gliosis and neurodegeneration in the cortex and hippocampus, thus preserving cognitive function.

Cellular senescence (CS) is a phenomenon in which cells stop proliferating in response to different stress stimuli, including oxidative stress, proteasome inhibition, autophagy malfunction, etc. Senescent cells are characterized by flattened and enlarged morphology and exhibit several molecular markers, including telomere-dysfunction-induced foci, senescence-associated heterochromatin foci (SAHF), lipofuscin granules, DNA scars, altered gene expression, absence of proliferative Ki-67 protein, the activity of senescence-associated β-galactosidase (SA-β-GAL), and the expression of tumor suppressors and cell cycle inhibitors (Watanabe et al., 2017; Yanagi et al., 2017; Dodig et al., 2019). They also show changes in gene expression, the absence of response to apoptotic and mitogenic stimuli, and metabolic reprogramming evidenced by the production of a secretome called “Senescence-Associated Secretory Phenotype” (SASP; Kuilman et al., 2010; Kong et al., 2011; Campisi, 2013; Salama et al., 2014). The SASP includes a variety of factors, such as interleukins, growth factors, chemokines, and metalloproteases. These factors can affect the surrounding cells by activating various cell surface receptors and signal transduction pathways (Coppé et al., 2010), creating a local tissue microenvironment that induces inflammation (Bianchi-Frias et al., 2010).

During obesity, the inflammation and oxidative stress generated in the brain increase the amount and accumulation of senescent cells, thus contributing to neuroinflammation due to the SASP secretion. The neuroinflammation induced by the senescent cells creates a vicious cycle that escalates inflammation and oxidative stress (Salas-Venegas et al., 2022). The components of SASP (proinflammatory and immunomodulatory cytokines) can interact with specific receptors, non-protein molecule factors, and exosomes (Gorgoulis et al., 2019). Thus, both soluble and insoluble interaction molecules are employed by senescent cells to influence the local microenvironment. However, with the accumulation of senescent cells during inflammation, its proinflammatory and potentially detrimental characteristics dominate and result in persistent inflammation and tissue damage (Nelke et al., 2022).

Two tumor suppressor pathways establish the CS, p53/p21, and p16INK4a/pRB, which can be used as proteins to validate the senescent state (McHugh and Gil, 2018). Animal models, mainly rodents, have been advantageous in studying obesity. Dietary manipulations through high-calorie intake are the most used for maintaining a more remarkable similarity with the establishment of obesity in humans (Von Diemen et al., 2006).

Notably, sexual differences have been reported concerning neurodegeneration and cognitive decline during aging (Santín-Márquez et al., 2021). Moreover, several neurological conditions are associated with sex differences in prevalence or outcome. For example, depression, multiple sclerosis, and Alzheimer’s are common in women (Hanamsagar and Bilbo, 2016). So, it is crucial to study pathogenetic mechanisms, their progression, the age of onset, and possible treatment response in female models to develop the correct interventions for women. Ignoring these differences could alter the meaning of the obtained results (Piscopo et al., 2021).

The objective of this study was to determine the effect of systemic inflammation produced by the chronic consumption of a hypercaloric diet on the establishment of senescence in the brain and its impact on cognitive deterioration in the obesity model of female Wistar rats.

## Materials and methods

### Chemicals

All chemicals and reagents were purchased from Sigma Chemical Co. (St. Louis, MO). The reagents obtained from other sources are detailed throughout the text.

### Animals

Sixty-four female Wistar rats (Rattus norvegicus) we used in this study. The animal was provided by the closed breeding colony at the Universidad Autónoma Metropolitana-Iztapalapa (UAM-I). They were housed four-per-cage in polycarbonate cages in a 12 h light–dark cycle and had free access to water and food. The animals’ health status was constantly evaluated. A good state of health was considered when the animals did not have tumors, skin, or ear infections and when they ate and drank properly. Rats with tumors and those that went blind were discarded from the study. All animal procedures were strictly carried out according to Mexican Official Ethics Standard NOM-062-ZOO-1999 and the Standard for the disposal of biological waste (NOM-087-ECOL-1995).

### Experimental groups

At 21 days of age, the 64 rats were randomly distributed into two groups: the Standard Diet (SD) group (*n* = 32) and the hypercaloric diet (HD) group (*n* = 32). The animals were euthanized at 13 months of age.

### Animal diets

HD diet was prepared following the protocols previously reported (Bautista et al., 2017; Toledo-Pérez et al., 2021). The HD is based on an obesogenic diet with 23.5% protein, 20% animal lard (40% saturated fats), 5% corn oil, (60.7% polyunsaturated fats, 24.3% monounsaturated fats, 15% saturated fats, 0% cholesterol), 20.2% polysaccharides, 20.2% of simple sugars, 5% of fiber, 5% of the mineral mix, and 1% of vitamins (caloric intake, 4.9 kcal/g). The rats started consuming HD after weaning (at 21 days old) until they were euthanized at 13 months of age.

The SD groups were fed an Abene BDL-7100 diet containing 23% protein, 4.5% fat, and 46.5% carbohydrates (caloric intake, 3.2 kcal/g). The weekly food consumption was measured, and the average was plotted monthly. The results represent the consumption per box divided by the number of animals per box.

### Morphometric and biochemical determinations

Animals were weighed and measured monthly to obtain the morphometric dimensions. The weight and naso-rectal length were used to obtain the Lee index to diagnose obesity in small animals, and those with an index greater than 0.30 were considered obese animals (Suárez Román et al., 2013). Rats were weighed with a digital scale 0SX40-SMART (Torrey, Mexico).

For the biochemical determinations, the blood (200 μl) was collected from the rats’ tail veins after a fast of 8 h at 6 and 13 months of age. Serum was obtained by centrifugation at 3500 rpm for 10 min. Subsequently, glucose, creatinine, triglycerides, cholesterol, GOT, GPT, GGT, and HDL were determined using a biochemical blood analyzer (Spotchem EZ SP-4430; Arkray Inc., Kyoto, Japan) and reactive strips: Spotchem II Kenshin-2, glu, and cre2 (Arkray Inc., Kyoto, Japan). The atherogenic index was calculated as AI = (Total Cholesterol – High-Density Lipoprotein HDL)/HDL (Othman et al., 2019).

#### Sample preparation for the enzyme-linked immunosorbent assay (ELISA assay)

Treated and control animals were euthanized at 6 and 13 months of age. The blood samples were collected and centrifuged (Eppendorf, Hamburg, Germany) to obtain the serum (3,500 rpm at 4° C for 15 min). The whole brain was carefully extracted and washed with saline solution. Brain cortex (Cx) and hippocampus (Hc) were dissected and homogenized in 500 μl of lysis buffer (20 mM Tris, 0.25 M sucrose, 2 mM EDTA, 10 mM EGTA, 1% Triton X-100) containing protease inhibitor (11,836,153,001, Roche Diagnostics, Indianapolis, IN, United States). The samples were centrifugated at 13,500 rpm at 4°C for 15 min to collect the supernatant for the ELISA and the Western blots assays (Ugalde-Muñiz et al., 2020). At maximum speed, the tissue was homogenized using a polytron PTMR2100 7,549 (Omni International).

### Cytokine and BDNF levels evaluation

TNFα, IL6, IL10, IL1β, MCP-1, and BDNF were measured in serum and brain tissue by sandwich ELISA assay following the provider’s instructions. TNF-α DuoSet ELISA (DY510), IL-6 DuoSet ELISA (DY506), IL-10 DuoSet ELISA (DY522), IL-1β DuoSet ELISA (DY501), MCP-1 DuoSet ELISA (DY3144), BDNF DuoSet ELISA (DY248), and DuoSet ELISA Ancillary Reagent Kit (DY008), all purchased from R&D Systems (Minneapolis, MN, United States). All required solutions were prepared with deionized water from a Milli-RQ system (Millipore, MA). Serum and brain protein were incubated for 18 h at 4° C with PBS-Tween20 (0.05%)/0.5% BSA, washed three times, and incubated with the corresponding detection antibody for 2 h at room temperature. Bound detection antibodies were detected using system a-HRP (avidin-HRP/Streptavidin-HRP) using TMB/H2O2 as the substrate. Optical density readings were at 450 nm (Epoch BioTek, Winooski, VT, United States). All assays were performed by duplicate (Ugalde-Muñiz et al., 2020).

### Novel object recognition test (NOR)

As reported previously, the NOR test was used to evaluate short-term and working memory in SD and HD groups at 6 and 13 months of age (Antunes and Biala, 2012; Santín-Márquez et al., 2021). The NOR test was performed using a 45 × 45 × 45 cm acrylic box. Each rat was introduced into the box for 5 min daily over three consecutive days as a training period. On the fourth day, a pre-test was performed. Two random objects with different geometric shapes were placed in the box, and the animals explored them for 5 min. The exploration time for each object was recorded. The objects were changed and cleaned with 70% vol/vol ethanol before and between use. On the fifth day, the test was performed by placing one of the objects previously presented along with a novel one. The animals were then allowed to explore for 5 min, and the interaction time and the number of interactions with the old and new objects were recorded. The Novel preference index was calculated by dividing the time spent exploring the novel object by the total exploration time multiplied by 100 to obtain a percentage value.

### Western blot analysis

Proteins (30 μg) from each fraction protein were separated on 12% SDS-PAGE, transferred to polyvinylidene difluoride membranes (Inmobilon-P, Millipore Billerica, MA), and incubated with specific primary antibodies against anti-βactin (sc-47,778), anti-γH2AX (sc-2,357), anti-p21 (sc-6,246), and anti-GLB (sc-65,670) dilution 1:1000. Membranes were washed three times with TBS-Tween and incubated with a horseradish peroxidase-conjugated secondary antibody dilution (1:1000; Santa Cruz Biotechnology, Santa Cruz, CA, United States) for 2 h. After three consecutive washes, the blots were developed using a commercial chemiluminescence reagent. The proportion of these proteins was quantified by densitometric analysis using Kodak Molecular Imaging Software (v.4.5.1).

### Brain section

Rats were perfused transcardially with 4% paraformaldehyde in PBS and drop-fixed in 4% paraformaldehyde for 24 h. The brains were rapidly dissected and immersed in PBS/30% sucrose for 24 h. The brains were washed with PBS and embedded in tissue-tek (4,583 Sakura finetek, Torrance, CA, United States). Brain coronal sections (24 μm) from the frontal cortex were mounted serially.

### SA-β-gal activity assay

The β-galactosidase activity was analyzed following the protocol described previously [26]. Brain sections were fixed with 4% paraformaldehyde, washed with PBS 1×, and stained with a solution containing 20 mg/ml of X-gal (V394A, Promega, Madison, WI, United States) in dimethylformamide, 0:2 M citric acid/sodium phosphate buffer pH = 6, 100 mM potassium ferrocyanide, 5 M sodium chloride, and 1 M magnesium chloride. Sections were incubated for 12 h at 37° C.

### Statistical analysis

All data were analyzed and graphed with Prism 8 (GraphPad Software). Specific tests were performed according to each experimental design and are indicated in each figure.

## Results

### Morphometric parameters

**Figure 1:**
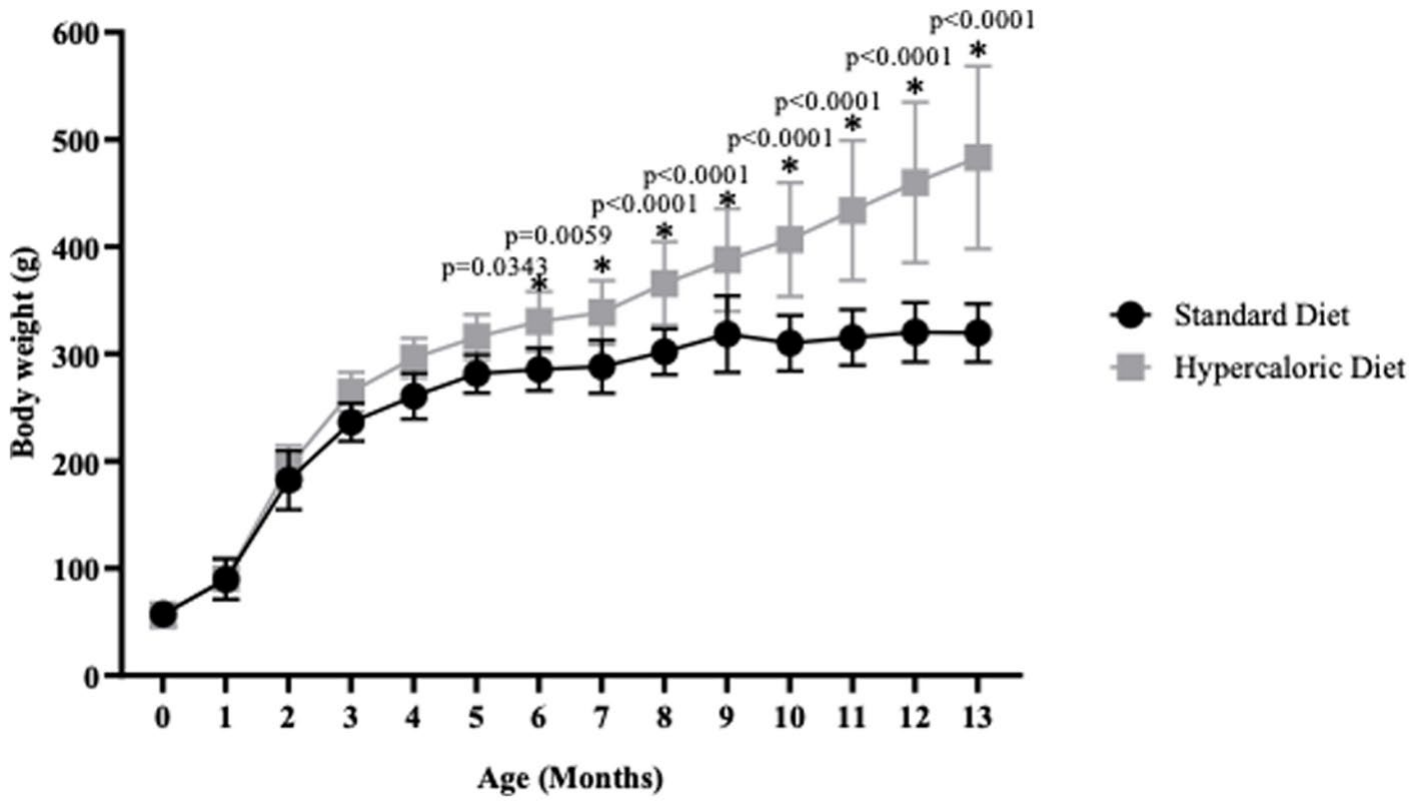
Body weight. Animal body weight was evaluated monthly (*n* = 17 SD and *n* = 15 HD). The data represent the mean ± standard deviation (SD) and were analyzed by ANOVA and *post hoc* Holm-Sidak. The *p*-values are indicated in the graph<0.05. The significant differences between groups compared to the HD are marked with *.

Figure 1 shows the obesity establishment in HD-fed rats. From month 6 of age, a 15% increase in body weight was observed in the HD group compared to the SD group (p < 0.034). The increment continued until the last evaluation at 13 months, when the HD group presented a 51% higher body weight than the SD group (p < 0.0001). Table 1 illustrates the naso-rectal length in both animal groups at 6 and 13 months. As expected, the length increased in both groups over time, with 6% growth in the SD group and 9% in the HD group. However, no significant differences were found between them, indicating that the physical development of the animals was similar regardless of the diet.

**Table 1:** Lee’s index.

Age	Group	Weight (g)	Lenght N-R (cm)	Lee’s index
6 months	SD	280.5 ± 16.96	22.65 ± 0.4408	**0.2889** ± 0.0080
	HD	361 ± 16.39	22.7375 ± 0.4897	**0.3132** ± 0.010*
13 months	SD	329 ± 27.23	23.95 ± 0.04870	**0.2878** ± 0.0050
	HD	532.25 ± 81.65	24.8 ± 1.150	**0.3262** ± 0.0189*

The data were evaluated in rats fed with SD and HD at 6 and 13 months of age (*n* = 10 SD *n* = 10 HD). The Lee index was obtained as described in the materials and methods section. The data represent mean ± SD and were analyzed by two-way ANOVA, followed by a Tukey’s *post hoc* test. The significant differences between groups compared to the SD are marked with *p < 0.05. The values in bold indicate the Lee´s index.

The Lee index was determined using the data of body weight and naso-rectal length. Table 1 shows that the Lee index in the HD group increased by 8% at 6 months of age and by 13% at 13 months of age compared to the SD group, confirming that HD animals were obese from 6 months of age.

### Food and water consumption

Food consumption was evaluated monthly (Supplementary Figure 1). In the third month, the SD group consumed 35% more food (measured in g) compared to the HD group, and from month 5 to month 10, there was an increase and then a decrease in food intake (32, 42, 36, 37, 35, and 25% respectively). However, as seen in Supplementary Figure 1B, the monthly kcal intake in the HD group was higher than in the SD group over time. The increase in calorie consumption in the HD group was 24% at month 4, and as time progressed, an increase of 22, 25, 29, and 26% was found at months 9, 10, 11, 12, and 13, respectively. Water consumption was also evaluated, but no differences were found between the groups (data not shown).

### Biochemical parameters

The biochemical parameters, including the GOT/GPT ratio, triglycerides, creatinine, glucose, cholesterol, high-density lipoprotein (HDL), atherogenic index, and low-density lipoprotein (LDL), are shown in Table 2. Some parameters significantly increased in the HD group compared to the SD group. In particular, an 11-fold increase in triglyceride levels was observed in the HD group at 13 months of age (86 mg/dl) compared to levels in the same group at 6 months (7.8 mg/dl). Augmented glucose levels were observed in both groups at 13 months of age compared to the values obtained at 6 months. A 1.30-fold increase in glucose levels was observed in the SD group and a 1.68-fold increase in the HD group. Regarding cholesterol levels, the SD group showed increased levels compared to the HD group at 6 months; however, at 13 months, the HD group increased their cholesterol levels by 1.40 times compared to the values obtained at 6 months.

**Table 2:** Biochemical parameters.

Parameter	6 months of age		13 months of age	
	SD	HD	SD	HD
GOT/GPT (IU/L)	2.669 ± 0.9436	7.651 ± 5.097	4.760 ± 0.9010	6.451 ± 1.515
TRIGLYCERIDES (mg/dl)	3.8 ± 5.495	7.8 ± 5.675	48.00 ± 17.65	86.00 ± 25.07 & *p* = 0.0229
CREATININE (mg/dl)	0.44 ± 0.2966	0.2 ± 0.07071	0.3400 ± 0.05477	0.3400 ± 0.05477
GLUCOSE (mg/dl)	88.2 ± 14.74	78.6 ± 10.69	115.4 ± 5.459 & *p* = 0.0080	132.8 ± 12.52 & *p* < 0.0001
CHOLESTEROL (mg/dl)	76.40 ± 9.317* *p* = 0.0298	56.60 ± 6.348	85.80 ± 12.50	79.60 ± 10.85 & *p* = 0.0108
HDL (mg/dl)	22.60 ± 6.107* *p* = 0.0029	8.800 ± 3.962	19.00 ± 6.519	14.40 ± 2.966
LDL (mg/dl)	53.04 ± 4.651	46.24 ± 3.648	57.20 ± 7.580	48.00 ± 7.331
ATHEROGENIC INDEX	2.569 ± 0.8980	6.270 ± 2.420* *p* = 0.0321	3.721 ± 0.7594	4.634 ± 0.8367

The table shows the values obtained of GOT/GPT, triglycerides, creatinine, glucose, cholesterol, HDL, LDL, and atherogenic index determined in rats fed with a standard diet (SD) and hypercaloric diet (HD) at 6 and 13 months of age (*n* = 5 SD and *n* = 5 HD). The data represent the mean ± SD and were analyzed by ANOVA. The significant differences between groups concerning the SD are marked with ∗, and differences at different ages are marked with & *p* < 0.05.

When evaluating the atherogenic index, a 2.4-fold increase was observed in the HD group compared to the SD group at 6 months. HDL concentrations were higher in the SD group compared to the HD group at 6 months of age. However, this parameter did not significantly change at 13 months of age.

It is essential to highlight that, despite finding differences in some evaluated parameters, none of these values were outside the clinical parameter limits established for Wistar laboratory rats.

### Inflammatory profile

#### Serum inflammatory profile

**Figure 2:**
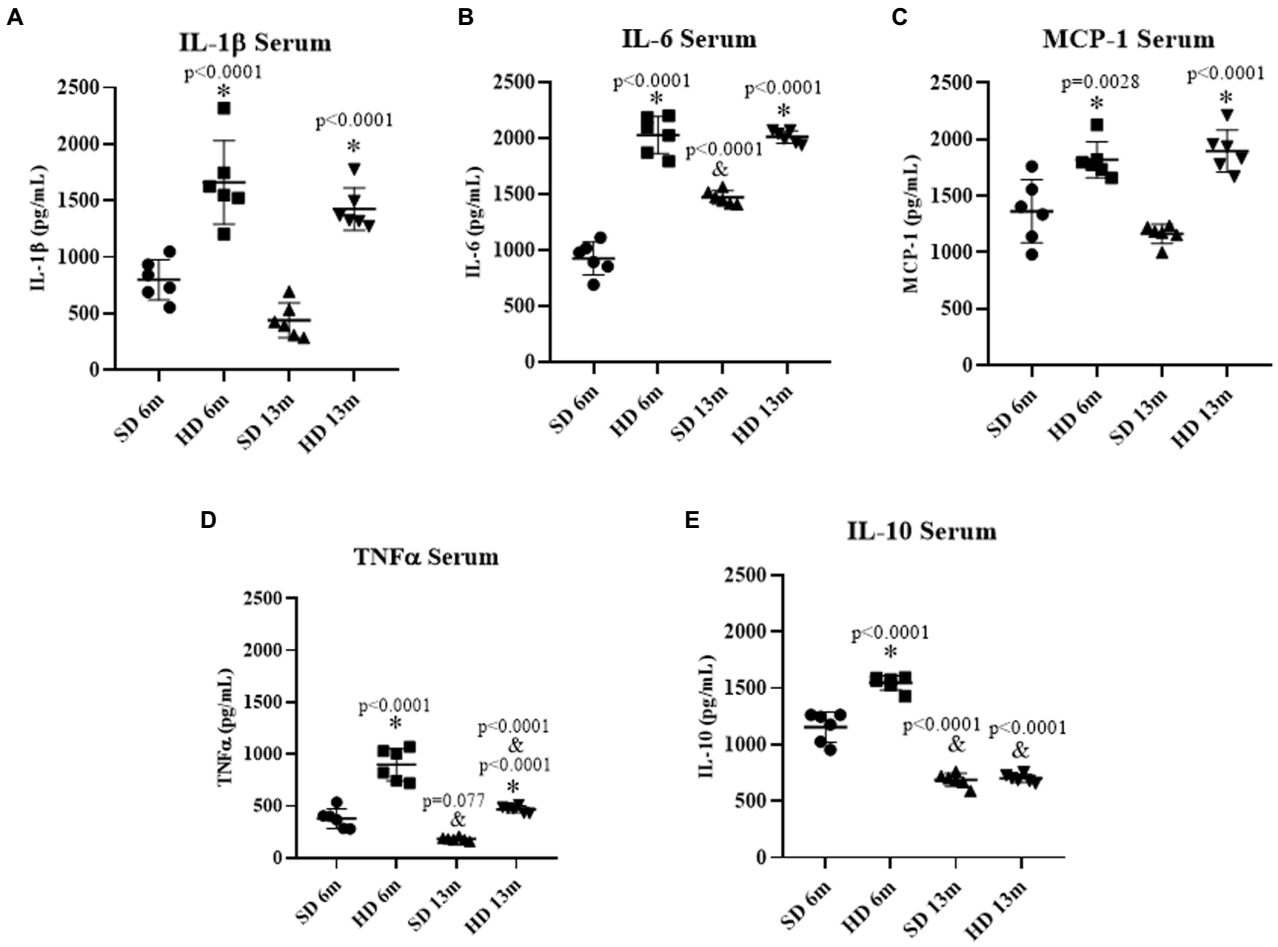
Serum inflammatory profile. The levels of IL1β **(A)**, IL6 **(B)**, MCP-1 **(C)**, TNFα **(D)**, and IL10 **(E)** were determined by ELISA (*n* = 6 SD and *n* = 6 HD). The data represent the mean ± standard deviation and were analyzed by two-way ANOVA, followed by Tukey’s *post hoc* test. The significant differences between groups with respect to the SD are marked with ∗, and differences at different ages are marked with & *p* < 0.05.

Once the obesity model was validated, the impact on the systemic inflammatory response was evaluated by quantifying the concentrations of the cytokines in the serum in both animal groups at 6 and 13 months of age. Figures 2A,B show that IL-1β and IL-6 levels in the HD group were significantly higher than in the SD group at 6 and 13 months of age; IL-1β levels were 2.07 times greater at 6 months and 3.23 times greater at 13 months in HD rats than in SD. While in the HD group, IL-6 levels increased 2.18 times at 6 months and 1.36 times at 13 months compared to the SD. Interestingly, in the SD group, an augment in this cytokine was also associated with age since it increased 1.58-fold at 13 months compared to 6 months.

The serum concentration of monocyte chemoattractant protein 1 (MCP-1) shown in Figure 2C presented the same behavior as IL-1β, both HD groups increased MCP-1 concentration compared to the SD groups (1.33 times at 6 months and 1.63 times at 13 months of age).

Figure 2D shows that TNF-α concentration in the HD group significantly increased by 2.36% at 6 months and 2.55% at 13 months of age compared to the SD group. However, both 13 moth-old groups decreased TNF-α concentration at month 13 of age. SD group significantly decreased by 2.06-fold and the HD group by 1.91-fold; the HF rats showed a higher TNF-α concentration.

IL-10 concentration was quantified to assess the anti-inflammatory response. Figure 2E shows that the HD group significantly increased by 1.33-fold at 6 months; however, at 13 months, IL-10 levels decreased in both groups, being more remarkable in the HD group (2.20 decrease fold) compared to the SD group (1.68-fold).

#### Cerebral cortex inflammatory profile

**Figure 3:**
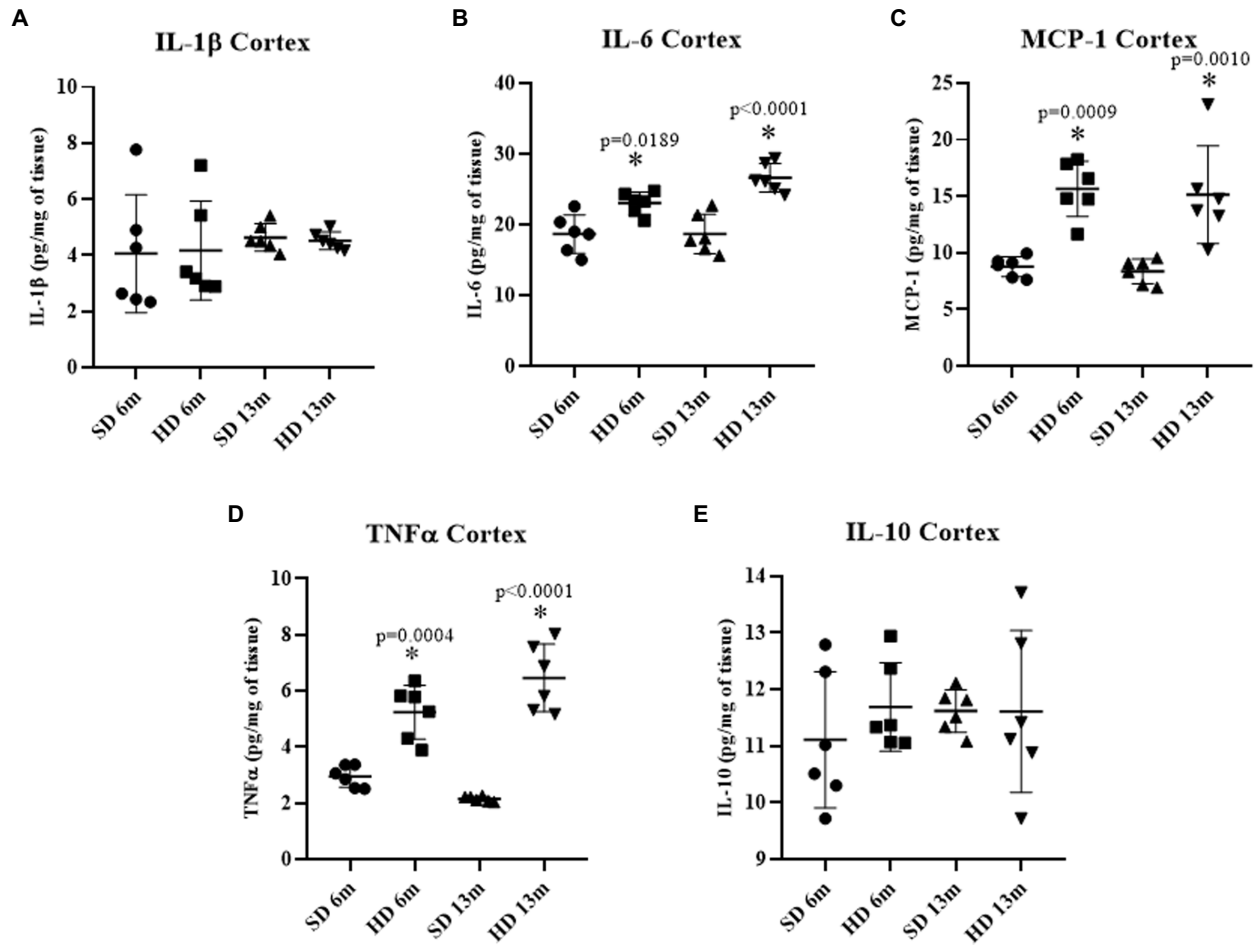
Cerebral cortex inflammatory profile. The levels of IL1β **(A)**, IL6 **(B)**, MCP-1 **(C)**, TNFα **(D)**, and IL10 **(E)** were analyzed by ELISA (*n* = 6 SD and *n* = 6 HD). The data represent the mean ± SD and were analyzed by two-way ANOVA, followed by Tukey’s *post hoc* test. The significant differences between groups compared to the SD are marked with ∗ *p* < 0.05.

Figure 3 shows cytokine concentrations in the rat cerebral cortex. No differences were found in IL-1β and IL-10 concentrations (Figures 3A,E), but a significant increase in IL-6 was found at the two-time points measured in the HD group compared to the SD group (Figure 3B). At 6 months of age, IL-6 concentration in the HD group was 1.23 times higher than in the SD group, and at 13 months, it was 1.42 times higher than in the SD group. MCP-1 levels showed the same behavior. The HD groups had significantly higher levels compared to the SD group (1.78-fold increase at 6 months of age and 1.81-fold at 13 months), suggesting that the HD group had higher Cx inflammation (Figure 3C). TNF-α levels in the HD group were significantly higher compared to the SD group at 6 months of age (1.77-fold increase) and 13 months of age (3.02-fold increase).

#### Hippocampus inflammatory profile

**Figure 4:**
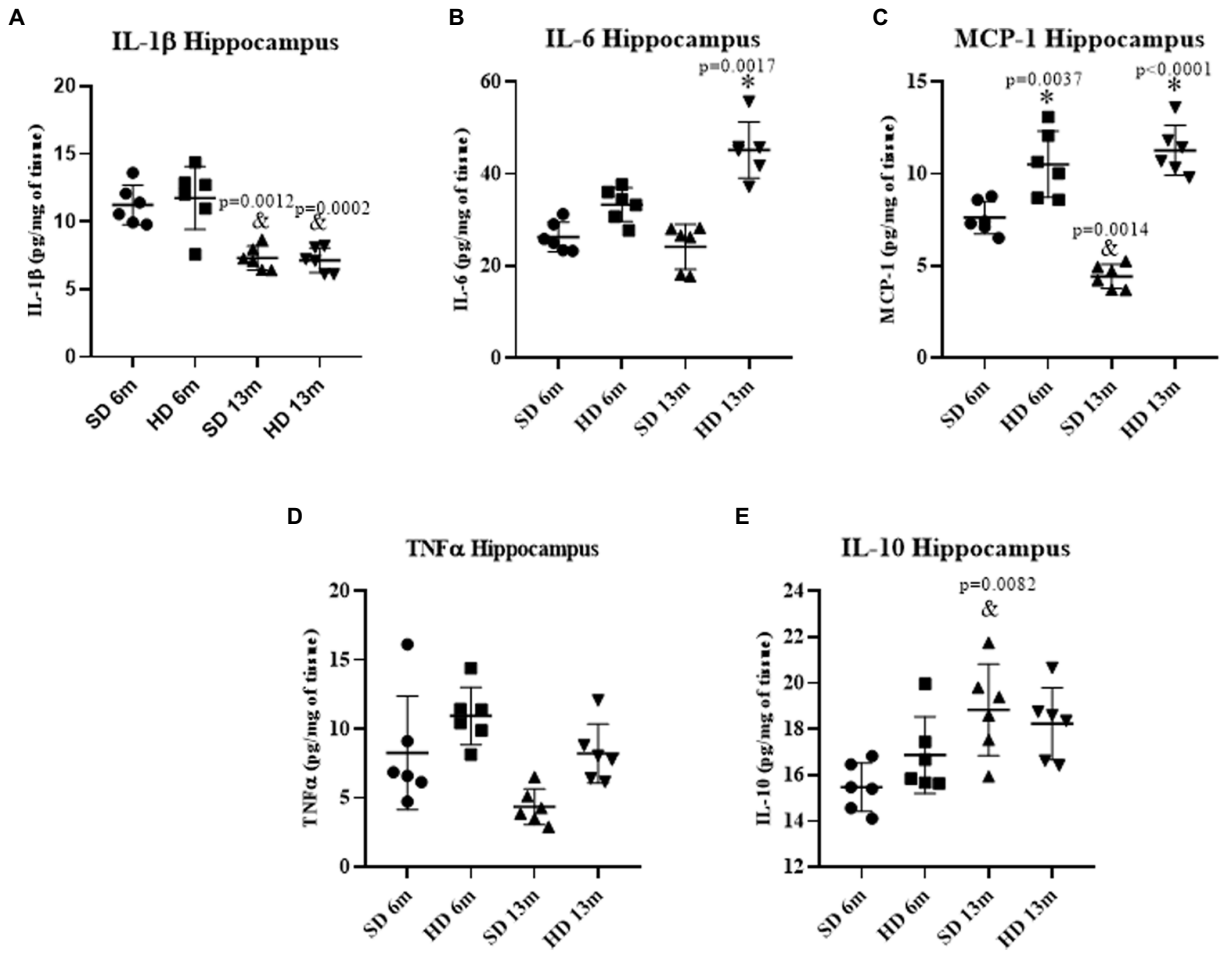
Hippocampus inflammatory profile. The levels of IL1β **(A)**, IL6 **(B)**, MCP-1 **(C)**, TNFα **(D)**, and IL10 **(E)** were analyzed by ELISA (*n* = 6 SD and *n* = 6 HD). The data represent the mean ± SD and were analyzed by two-way ANOVA, followed by a Tukey’s *post hoc* test. The significant differences between groups compared to the SD are marked with ∗, and differences at different ages are marked with & *p* < 0.05.

Figure 4A shows a significant decrease in hippocampal IL-1β concentrations in both study groups (SD and HD) at 13 months of age, but no differences were observed regarding HD diet consumption. IL-6 concentration did not change at 6 months either (Figure 4B), but an increase was found at 13 months of age in the HD group (1.86-fold more than the same age SD group and the young groups). While MCP-1 concentrations in the HD group (Figure 4C) significantly increased at 6 months of age (1.37-fold) and 13 months of age (2.54-fold) compared to the SD group. A decrease in the concentration of MCP-1 was observed in the SD group at 13 months of age compared to the younger group (1.72-fold). No significant differences were found in TNF-α and IL-10 concentrations (Figures 4D,E) between the two groups at 6 and 13 months. However, a 1.27-fold increase in IL-10 concentrations was observed in the SD group at 13 months compared to the 6 months group.

### Brain-derived neurotrophic factor (BDNF)

**Figure 5:**
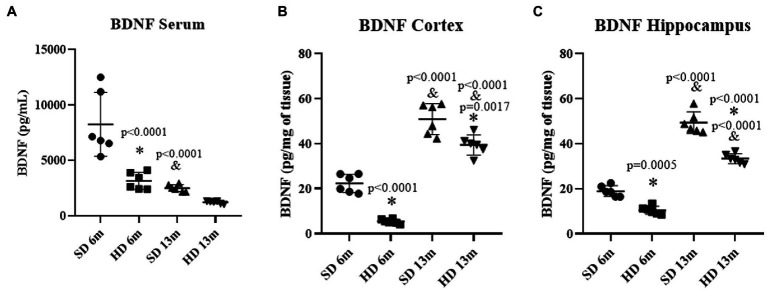
Brain Derived Neurotrophic Factor levels. The levels of BDNF in serum **(A)**, cortex **(B)**, and hippocampus **(C)** were determined by ELISA (*n* = 6 SD and *n* = 6 HD). The data represent the mean ± SD and were analyzed by two-way ANOVA, followed by Tukey’s *post hoc* test. The significant differences between groups compared to the SD are marked with ∗, and differences at different ages are marked with & *p* < 0.05.

Figure 5A shows that serum BDNF concentration decreased by 2.61 times in the HD group at 6 months of age compared to the SD group at the same age. A 3.28-fold decrease in BDNF concentration was also observed in the SD group at 13 months compared to the values obtained at 6 months in this same group. Concerning BDNF levels in the Cx (Figure 5B), a 4.1-fold decrease was observed in the HD group compared to the SD group at 6 months. However, at 13 months, BDNF increased in both groups (SD and HD) compared to the levels found at 6 months. In the Hc (Figure 5C), BDNF concentrations diminished in the HD group compared to the SD group at 6 and 13 months. In summary, BDNF increased in both groups (SD and HD) at 13 months, as opposed to 6 months.

### Novel object recognition test (NOR)

**Figure 6:**
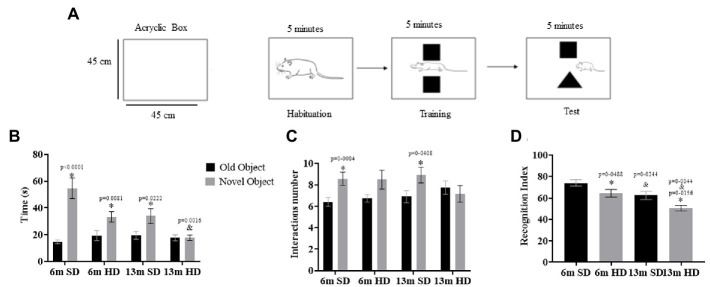
Novel object recognition test. **(A)** Schematic representation of protocol for a novel object recognition test. Familiar and novel object interaction time **(B)**, interactions number **(C)**, and recognition index **(D)** determined in rats fed with SD and HD at 6 and 13 months of age (*n* = 12 SD and *n* = 12 HD). Each bar represents the mean ± SD. The data were analyzed by two-way ANOVA and Tukey’s *post hoc* test. The significant differences between groups with respect to the SD are marked with ∗, and differences at different ages are marked with & *p* < 0.05.

The NOR test was performed to determine the possible detrimental effects of neuroinflammation on cognition; the NOR test was performed at 6 and 13 months of age following the scheme shown in Figure 6A. The interaction time with the novel object is shown in Figure 6B; the rats in the SD and HD groups spent more time exploring the novel object at 6 months than at 13. However, at 13 months, the SD group did not differentiate between the familiar and the novel object, equaling the exploration time between the objects.

When evaluating the number of interactions with each object, the SD group at 6 and 13 months interacted similarly with both objects (Figure 6C). With an evident decrease in the recognition index, the HD rats did not discriminate the novel object from the familiar object at 6 months, as seen in Figure 6D, being more pronounced in both groups at 13 months of age, especially in the HD group, which presented a more significant deficit in recognizing novel objects.

### Senescence markers

**Figure 7:**
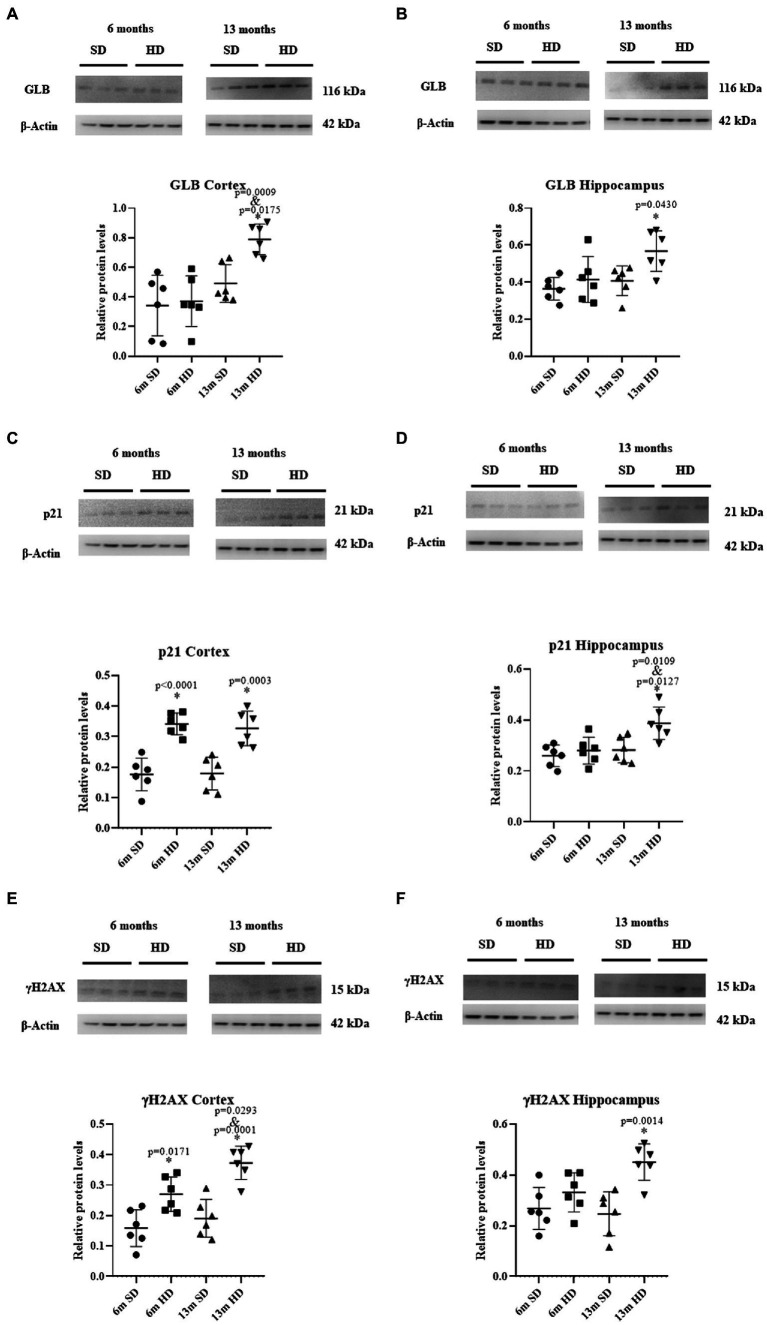
Expression of molecular markers of senescence in brain. Representative Western blot analysis of GLB in the Cx **(A)** and Hc **(B)**; p21 in the Cx **(C)** and Hc **(D)**; and γH2AX in the Cx **(E)** and Hc **(F)**. The graphs show the densitometric protein expression analysis from six animals per age group (each dot represents an animal; *n* = 6 SD and *n* = 6 HD). The data represent the mean ± SD, the data were compared using the two-way ANOVA and Tukey’s *post hoc* test. The significant differences between groups compared to the SD are marked with *, and differences at different ages are marked with & *p* < 0.05.

To evaluate the participation of senescence in cognitive deterioration, GLB, p21, and γH2AX were determined in the Cx and Hc at 6 and 13 months of age. Figure 7A shows a significant increase of 1.12 times in GLB expression in the Cx of the 13-month-old HD group compared to the SD group at the same age. Unlike the SD group, where no changes in the expression of this enzyme were observed throughout life, changes were observed in the HD group at 13 months of age, with an increment of 2.12 times compared to 6 months. No differences were observed in GLB expression in this region at 6 months old, regardless of the diet.

As observed in Figure 7B, GLB expression increased in the Hc at 13 months of age in rats in the HD group compared to the SD group. At 6 months, no differences in GLB expression were observed in this brain region between the SD and HD groups. Figure 7C indicates that the HD group had significantly higher levels of p21 in the Cx compared to the SD group at 6 months and 13 months, with an increase of 1.93 and 1.82-fold, respectively. The p21 levels in the SD group were low and stable, while in the HD group, they were high and constant. No differences were obtained in p21 expression in the Hc at 6 months of age (Figure 7D) between the SD and HD groups. However, at 13 months of age, a significant increase in p21 levels in the Hc was observed in the HD group compared to the SD group (1.38-fold). In addition, the same increase in p21 levels was found in the HD group at 13 months compared to the 6 months in the same group. Figure 7E shows that the expression of histone γH2AX in the Cx of HD rats increased 1.70 and 1.95 times compared to the SD group at 6 and 13 months, respectively; the levels of this histone increased in the HD group at 13 months compared to the 6 months old rats. When evaluating the expression levels of γH2AX in the Hc (Figure 7F), a significant increase of 1.82 times was found in the HD group at 13 months of age compared to the SD group. No changes in the expression of this histone were found at 6 months between both groups.

### SA-β-gal assay

Senescent cells in the Cx and Hc were only evaluated at 13 months in SD and HD groups because we only found changes in all the senescence markers at this age.

**Figure 8:**
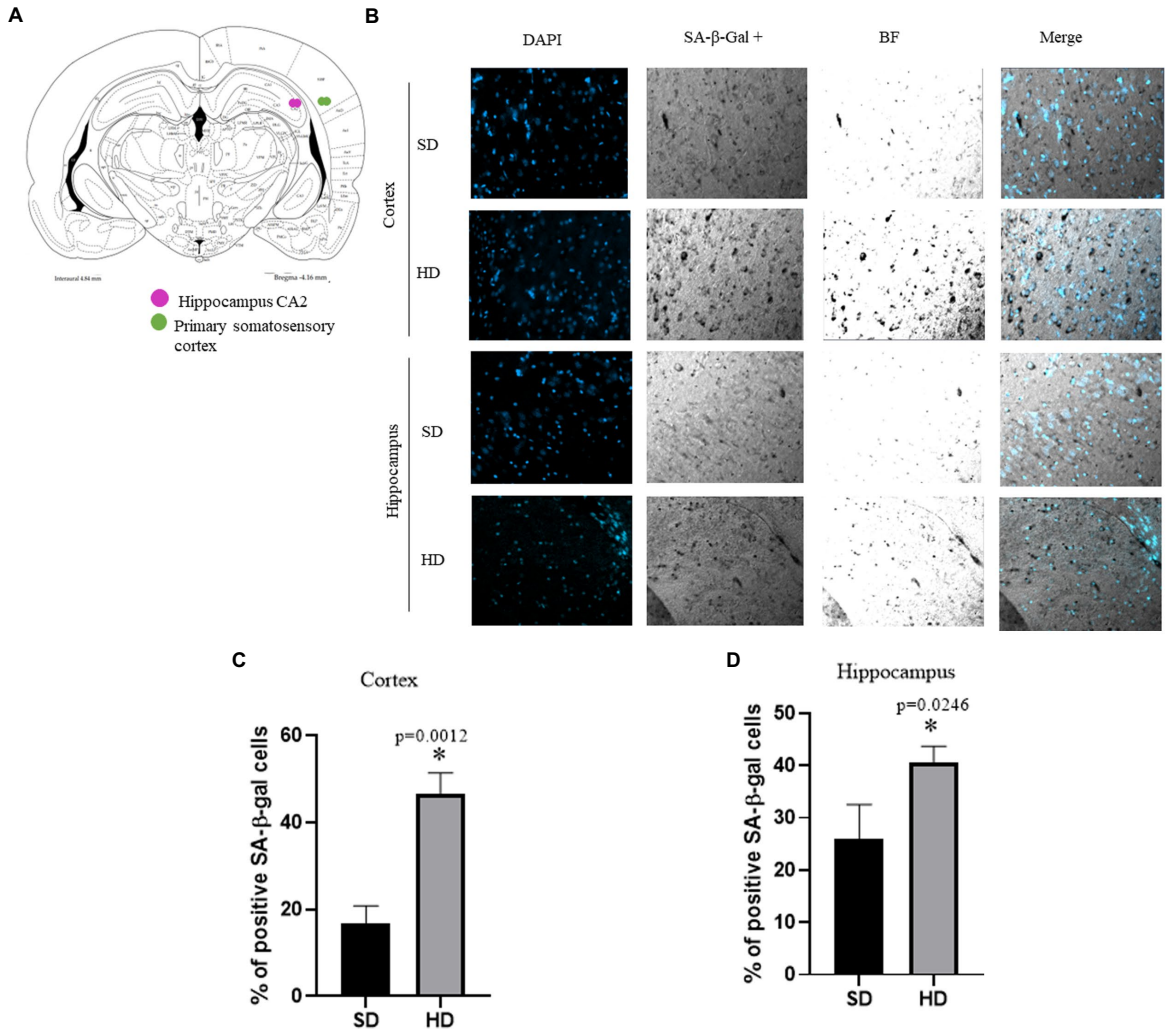
Evaluation of senescent cells in brain. **(A)** Anatomic diagram to locate the regions analyzed. **(B)** Representative images of different regions with cells with senescence features accumulate in the Cx and Hc in animals with HD at 13 months (*n* = 6 SD and *n* = 6 HD). The percentage of SA-β-gal positive cells per area in each brain section in cortex **(C)** and hippocampus **(D)**. The average of three brains per group is graphed. The data represent the mean ± SD and were analyzed by two-way ANOVA, followed by Tukey’s *post hoc* test. The significant differences between groups compared to the SD are marked with ∗ and differences at different ages are marked with & *p* < 0.05. Confocal fluorescence micrographs are 20× magnification. *n* = 3.

Figure 8A is an anatomical diagram illustrating the specific regions of the brain in which the presence of senescent cells was studied. The CA2 region of the hypothalamus and the region of the somatosensory cortex are specifically mentioned. These regions are essential for memory and sensory perception, respectively. The lysosomal β-galactosidase assay was used to detect the presence of senescence in these brain areas. Figure 8B shows SA-β-gal positive cells in Hc (CA2 specifically) and Cx (somatosensory cortex) in rats with SD and HD at 13 months of age. The presence of senescent cells is more evident in the HD group in both areas analyzed in concordance with the increase of senescent markers previously shown.

Figures 8C,D show the quantification of the SA-β-gal positive cells in the Cx and Hc. In the HD group, there was an increase of 30% in SA-β-gal positive cells in the Cx and 14% in the Hc compared to the SD group. These results suggest a higher number of senescent cells in subjects in the HD group compared to those in the SD group.

## Discussion

Obesity is a complex condition that affects individuals both physically and mentally. Physically, it increases the risk of chronic diseases such as diabetes, cardiovascular disease, cognitive decline, and certain cancers. At a psychological level, it can lead to low self-esteem, depression, and decreased quality of life. Our study focused on understanding the link between obesity and cognitive decline during long-term HD in middle-aged female rats since there is a higher rate of obesity in women, and they have a greater propensity to present dementia during advanced age (Albanese et al., 2015; Ghasemi Yngyknd et al., 2021).

The weight gain and the Lee index of the HD rats concord with previous studies (Malafaia et al., 2013; Suárez Román et al., 2013; Toledo-Pérez et al., 2021, p. 23), thus validating the obesity model. Moreover, we also found an increase in the atherogenic index, a risk marker for atherosclerosis and coronary heart disease (Niroumand et al., 2015), in the HD groups at 6 and 13 months of age. Interestingly, the SD group showed an increase in cholesterol and HDL levels, which could be due to the effect of reverse cholesterol transport, as described by Marques et al., 2018, as a mechanism for removing excess cholesterol from peripheral tissues and redistributing it or removing it from the body by the gallbladder.

At 13 months of age, both groups presented an increase in triglycerides, glucose, and cholesterol, which could be due to the transition from young adulthood to middle age, generating an accumulation of excess visceral fat, muscle mass, and strength loss, and an increase in adipocyte size in subcutaneous fat leading to increased release of free fatty acids (FFAs) associated with insulin resistance and other metabolic abnormalities, as reported by Palmer and Jensen, 2022. It is important to note that no significant differences were found when comparing the values of these parameters between the two groups at 6 and 13 months considering the Harlan manual (Giknis and Clifford, 2008).

Our results agree with previous findings, where obesity-induced chronic low-grade inflammation (Bing, 2015; Sindhu et al., 2015; Khanna et al., 2022) generates an increase in MCP-1, IL-1β, and IL-6 as observed in the HD group at 6 and 13 months. Serum levels of these cytokines are raised in obese individuals and those with chronic inflammatory conditions and abnormal serum lipid concentrations (Panee, 2012; Sindhu et al., 2015; El-Mikkawy et al., 2020), which aligns with the changes in the biochemical profile observed in our study (shown in Table 2). Additionally, IL-6 adversely impacts lipid metabolism by increasing triglyceride release and reducing lipoprotein lipase activity (Werida et al., 2021). Interestingly, we found an increase in the circulating levels of TNF-α in the HD groups at 6 and 13 months of age; however, those levels decreased at 13 months of age. This observation could be explained due to the individual immune system responsiveness and adaptability to a particular inflammatory condition regarding the degree of stimulation (severity) and persistence (chronicity; Italiani et al., 2020). The same behavior was observed for the anti-inflammatory cytokine IL-10, which increased in HD rats at 6 months and decreased in both groups at 13 months. This phenomenon is consistent with other studies that have reported that IL-10 decreases with age and obesity in animal and human models (Dagdeviren et al., 2017; Kondo et al., 2018), especially in obese women (Esposito et al., 2003).

Low-grade systemic inflammation induced by obesity also affects the central nervous system (CNS), generating neuroinflammation mainly in the Hc and Cx, impairing learning and memory (Henn et al., 2022). Accordingly, we found elevated cytokine levels in those brain regions in the HD groups. IL-6, TNF-α, and MCP-1 levels increased in the Cx of HD-rats at 6 and 13 months of age (Figures 3B–D), while in the Hc, we found a rise in IL-6 in the HD group only at 13 months, and MCP-1 at 6 and 13 months. The cytokine increment indicates an ongoing inflammatory response in these brain regions, which may contribute to developing neurological disorders and learning deficits (Schmitt and Gaspar, 2023). Since the Cx has a higher number of neurons and fewer astrocytes and microglia than the Hc, these differences in cell composition could contribute to the observed differences in response to HD and obesity.

Moreover, obesity-induced inflammation changes the blood–brain barrier integrity, leading to increased permeability and the entrance of proinflammatory cytokines to the brain, which activates glial cells, such as microglia and astrocytes, leading to the activation of these cells (Salas-Venegas et al., 2022). This process can contribute to the harmful effects of obesity on the brain. Activated microglia secrete more proinflammatory cytokines, such as TNFα, IL-1β, and IL-6, which can perpetuate neuroinflammation and result in neuronal damage. Additionally, reactive astrocytes activated by proinflammatory signals have been linked to synaptic degeneration and glutamate dysregulation, contributing to neurodegeneration (Kwon and Koh, 2020). These mechanisms highlight the complex interplay between obesity-induced inflammation and brain function.

Brain-derived neurotrophic factor (BDNF) is a central molecule in neuronal outgrowth, differentiation, repair, and synaptic connections. Its levels are tightly regulated in response to different stimuli (Sandrini et al., 2018). Here a decrease in BDNF circulating levels in the HD group at 6 months of age was found, which further decreased with age. This observation is consistent with other studies that have found lower levels of BDNF in obese organisms. Reduced levels of BDNF can impact neural development and cognitive function by diminishing neuroplasticity, which is essential for learning, memory, and other cognitive functions. In rodents and humans, high levels of BDNF have been detected in various regions of a normal brain, including the hippocampus, amygdala, cerebellum, and cortex (Miranda et al., 2019).

Moreover, BDNF increases in the murine Hc during normal aging but not in pathological aging (Halbach, 2010). Here, lower BDNF levels in the Cx and Hc were determined in the HD group at 6 months. According to the literature, higher BDNF levels were determined at 13 months of age in the Cx and Hc of both animals groups when compared to the 6 months old rats; however, the levels observed in the HD group were significantly lower than the SD group in both brain regions, suggesting that BDNF decreased levels could contribute to the learning and memory deficits observed in obese individuals. Our results seem to align with previous studies that have reported a negative correlation between serum BDNF levels, obesity markers, and inflammation (Raharjo et al., 2021).

Research on animal models of obesity has suggested a link between obesity-induced inflammation and the development of cognitive alterations (Schmitt and Gaspar, 2023). Our results using the NOR test showed that rats in the HD group significantly decreased their ability to discriminate between the new and the familiar object at 6 months. That was more evident at 13 months of age. Interestingly, rats in the SD group also diminished their ability to discriminate the objects with age.

These results suggest that both diet-induced obesity and normal aging can impair the ability to discriminate a new object, which is an indicator of hippocampal-dependent learning and memory. The decline in performance could be due to the negative impact of obesity on the integrity of the Hc and other brain regions, such as the Cx, as well as the changes in synaptic plasticity and dendritic spine density associated with neuroinflammation and BDNF dysregulation. These findings highlight the importance of considering obesity and aging as risk factors for cognitive decline.

The onset of obesity is related to increased senescent cells within adipose tissue and other organs in response to various stressful stimuli (Chaib et al., 2021). Thus, the SASP is increasingly recognized as a critical driver of age-related pathologies, such as chronic inflammation. Of note, SASP components are effective proinflammatory cytokines capable of attracting and activating immune cells, thereby promoting an inflammatory tissue environment. Thus cells with a senescent phenotype resistant to apoptosis might provide a proinflammatory microenvironment sustaining the progression of neuroinflammatory diseases (Nelke et al., 2022).

Cellular senescence is a process in which cells change their characteristic phenotype in response to stress and enter a state of prolonged cell cycle arrest accompanied by a distinctive secretory phenotype which can alter the tissue microenvironment and contribute to inflammation, oxidative stress, and tissue dysfunction (Tchkonia et al., 2013; Rachmian and Krizhanovsky, 2022). It is known that senescent cells accumulate during obesity (Minamino et al., 2009; Ogrodnik et al., 2017; Schafer et al., 2017).

Senescent cells in the tissues contribute to chronic inflammation in two ways. First, additive or synergistic effects of paracrine senescence amplify the SASP and exacerbate inflammation. Mediators of paracrine senescence include reactive oxygen species (ROS) signaling through gap junctions, growth factors and chemokines, and small extracellular vesicles. Second, the impaired immune function and a reduced rate of senescent cell clearance. The resulting accumulation of senescent cells further reinforces the inflammation (Guerrero et al., 2021).

In a study realized by Ogrodnik and collaborators, they found that obesity results in the accumulation of senescent glial cells in proximity to the lateral ventricle, a region in which adult neurogenesis occurs, their elimination restored neurogenesis and alleviated anxiety-related behavior (Ogrodnik et al., 2017). We decided to evaluate the expression of senescence markers (GLB, p21, and γH2AX) in middle age rats (13 months old) during obesity. We found a significant rise in the three senescence markers at 13 months in the HD group in the Cx and Hc. In recent years, evidence emerged supporting the accumulation of senescent cells in the brain during several neurological disorders. This data correlated with an increase in the number of cells SA-β-gal positive cells in these brain regions. Some studies showed that the accumulation of senescent glial cells and neurons leads to structural and functional changes in the brain that result in cognitive impairment in the context of aging (Sikora et al., 2021). Hence, our study’s contribution is that we indeed demonstrate that obesity-related cellular senescence can contribute to the development of age-related cognitive decline. The increased expression of senescence markers in the HD group reveals a higher burden of senescent cells in the Cx and Hc of obese rats, which may contribute to the observed decline in cognitive function. Their clearance has been shown to delay and alleviate those neurodegenerative symptoms (Kirkland and Tchkonia, 2017). Further research is needed to understand the exact mechanisms by which senescent cells contribute to cognitive decline in obesity. Targeting senescence is a promising strategy to prevent obesity-related neuropsychiatric diseases.

## Data availability statement

The raw data supporting the conclusions of this article will be made available by the authors, without undue reservation.

## Ethics statement

The animal study was reviewed and approved by COMISIÓN ACADÉMICA DE ETICA DE LA DIVISIÓN DE CIENCIAS BIOLÓGICAS Y DE LA SALUD. Dictamen: CECBS21-02.

## Author contributions

VS-V was involved in the design of the study, generation, collection, and interpretation of the data, as well as in the manuscript writing. RS-M, RR-C, YR-C, and AC-M assisted in the generation, collection, and interpretation of the data. AL-L and AC were involved in the analysis and interpretation of the data. VS-V, MK, and NL-D were involved in the design of the study, analysis of the data, and revision of the manuscript. MK and NL-D also supervised the investigation. All authors contributed to the article and approved the submitted version.

## Funding

This work was supported by the Consejo Nacional de Ciencia y Tecnología (CONACyT) grant FORDECYT-PRONACES/263957/2020, PRODEP UAM-PTC-695, and CONACYT Ciencia de Frontera 2019 (1783), as well as Dirección General de Asuntos del Personal Académico, Universidad Nacional Autónoma de México (UNAM), PAPIIT (IN214821). VS-V, RS-M, RR-C, and YR-C are CONACyT scholarship holders.

## Conflict of interest

The authors declare that the research was conducted in the absence of any commercial or financial relationships that could be construed as a potential conflict of interest.

## Publisher’s note

All claims expressed in this article are solely those of the authors and do not necessarily represent those of their affiliated organizations, or those of the publisher, the editors and the reviewers. Any product that may be evaluated in this article, or claim that may be made by its manufacturer, is not guaranteed or endorsed by the publisher.
