# Supplementary figures and images for: Chronic consumption of a hypercaloric diet increases neuroinflammation and brain senescence, promoting cognitive decline in middle-aged female Wistar rats
Source: Front Aging Neurosci. 2023 Apr 17;15:1162747. doi: 10.3389/fnagi.2023.1162747 (PMC10149996; doi:10.3389/fnagi.2023.1162747)

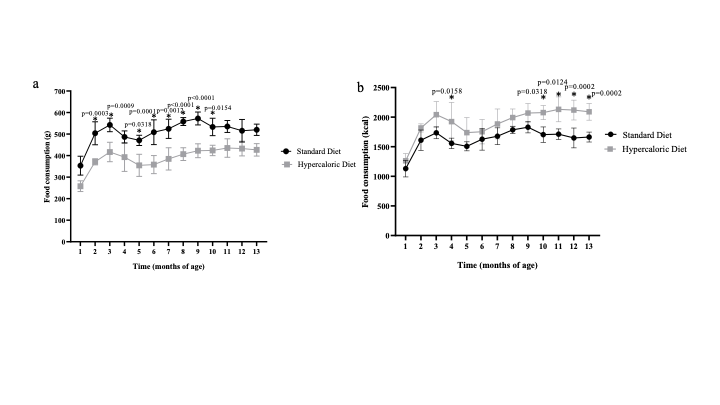

Supplement: Supplementary file 1 [file Image_1.TIFF]
